# Inactivation of the *Celf1* Gene that Encodes an RNA-Binding Protein Delays the First Wave of Spermatogenesis in Mice

**DOI:** 10.1371/journal.pone.0046337

**Published:** 2012-10-02

**Authors:** Marie Cibois, Gaella Boulanger, Yann Audic, Luc Paillard, Carole Gautier-Courteille

**Affiliations:** 1 Université de Rennes 1, Université Européenne de Bretagne, Biosit, Rennes, France; 2 Institut de Génétique et Développement de Rennes, CNRS UMR6290, Rennes, France; Leibniz Institute for Age Research - Fritz Lipmann Institute (FLI), Germany

## Abstract

**Background:**

The first wave of spermatogenesis in mammals is characterized by a sequential and synchronous appearance of germ cells in the prepubertal testis. Post-transcriptional controls of gene expression play important roles in this process but the molecular actors that underlie them are poorly known.

**Methodology/principal findings:**

We evaluated the requirement for the RNA-binding protein CELF1 during the first wave of spermatogenesis in mice. Mice inactivated for *Celf1* gene were not viable on pure genetic backgrounds. On a mixed background, we observed by histology and gene profiling by RT-qPCR that the testes of inactivated prepubertal mice were characterized by several features. (i) Spermiogenesis (differentiation of post-meiotic cells) was blocked in a subset of prepubertal inactivated mice. (ii) The appearance of the different stages of germ cell development was delayed by several days. (iii) The expression of markers of Leydig cells functions was similarly delayed.

**Conclusions/significance:**

*Celf1* disruption is responsible for a blockage of spermiogenesis both in adults and in prepubertal males. Hence, the spermiogenesis defects found in *Celf1*-inactivated adults appear from the first wave of spermiogenesis. The disruption of *Celf1* gene is also responsible for a fully penetrant delayed first wave of spermatogenesis, and a delay of steroidogenesis may be the cause for the delay of germ cells differentiation.

## Introduction

Mammalian spermatogenesis is a complex process that can be divided into three stages. During the pre-meiotic stage, spermatogonia divide actively by mitosis. The cells entering the meiotic phase are named spermatocytes. It is during the meiotic phase that recombinations occur, more specifically in pachytene spermatocytes [1]. Finally, the post-meiotic phase, or spermiogenesis, is characterized by deep morphological and structural modifications of germ cells that transform round spermatids into elongated spermatids and finally spermatozoa [2].

This complex differentiation process requires stage-specific expression of several gene products, and relies on tightly controlled gene expression. A prerequisite to understand these regulations is to characterize the stage-specific transcriptomes of germ cells. This was achieved by microarray analysis starting from enriched germ cell populations [3], but also starting from different ages of prepubertal testes. In mice, the first wave of spermatogenesis in prepubertal males extends over a period of 35 days after birth [4] and corresponds to the appearance of each germ cell stage within seminiferous tubules in a sequential manner. Therefore, whereas spermatozoa are produced asynchronously during postpubertal life, germ cells differentiate synchronously during the first wave of spermatogenesis. Hence, prepubertal testes of a given age have homogeneous contents, and comparing the transcriptome contents of gonads at different ages allowed the identification of stage-specific transcripts [5], [6], and putative transcriptional networks were proposed from these data [7].

During spermatogenesis, there exist two stages when transcription is blocked, in pachytene spermatocytes and during the late steps of spermiogenesis (reviewed in [8]). Hence, post-transcriptional regulations (controls exerted on pre-mRNAs or mRNAs) are probably particularly important. Indeed, high-throughput analyses of gene expression have highlighted adult testis as one of the organs with the highest level of alternative splicing events [9], [10]. In the developing testis, more than 700 mRNAs are translationally regulated [11], but very little is known about the molecular actors of the post-transcriptional controls active during the first wave of spermatogenesis.

The goal of the present work was to evaluate if the RNA-binding protein CELF1 was required for the first wave of spermatogenesis in mice. CELF1 (CUGBP1 and ETR3 like factor 1, also named CUGBP1 or EDEN-BP) is a member of the vertebrate CELF family of RNA-Binding proteins that play several roles in post-transcriptional controls. In the nucleus, it regulates alternative splicing by stimulating either the inclusion or the skipping of non constitutive exons. In the cytoplasm, it regulates the translation and stability of bound mRNAs [12], [13], [14]. Xenopus CELF1 binds to the 3′ untranslated region (3′UTR) of certain mRNAs via specific sequence elements leading to the rapid deadenylation, destabilisation and translational repression of these mRNAs [15], [16], [17]. In a previous study [18], we showed that *Celf1* inactivated mice had growth, viability and fertility defects. In males, hypofertility or sterility is associated with defects of spermiogenesis that can reach a complete blockage at stage 7 of round spermatids [18]. In the present article, we analysed the first wave of spermatogenesis in *Celf1* inactivated mice to test if the spermiogenesis defects in adults arise from a defective maintenance of spermiogenesis or are set up during prepubertal life. We found that the inactivation of *Celf1* hampers spermiogenesis in prepubertal animals like is adults, but also delays the first wave of spermatogenesis at both the germ cells and Leydig cells levels.

## Results

### The Disrupted Allele of *Celf1* is not Viable on 129SvPas Nor C57BL/6N Backgrounds

We have previously shown that the sterility phenotype of *Celf1*-inactivated mice (*Celf1* ^tm1Cba/tm1Cba^, hereafter *Celf1* ^−/−^ or −/−) was not fully penetrant. In males, 5/15 and 4/15*−/−* mice were respectively completely sterile and fully fertile, while the remaining 6/15 males had intermediate fertilities. This was attributed to the mixed genetic background of the mice [18]. In an attempt to fix that variability, we transferred the disrupted allele of *Celf1* on 129SvPas and C57BL/6N genetic backgrounds. We next crossed heterozygous mice (*Celf1* ^+/−^, +/−). We frequently found dead newborns that all had a −/− genotype; by contrast, none of the pups that were alive at 8–10 days post-partum (dpp) had that genotype (Table 1). Taking into account all the born pups (live and dead), the proportion of −/− animals did not differ from the expected Mendalian ones, which does not support a hypothetical prenatal morbidity. Hence, homozygous *Celf1* ^−/−^ mice on pure 129SvPas or C57BL/6N backgrounds die within the first day after birth. Consequently, we made the following experiments with the same mixed 129SvPas*C57BL/6N background as previously described [18].

**Table 1:** Homozygous Celf1^−/−^ mice are not viable on 129SvPas nor C57BL/6N pure backgrounds.

Background	Number of females	Number of litters	Number of live pups		Number of dead pups		Likelihood of Mendeliansegregation (P-value),live pups only	Likelihood of Mendelian segregation (P-value), all pups
			−/−	other	−/−	other		
129SvPas	6	12	0	51	12	0	3.7e-05	0.27
C57BL/6N	3	6	0	26	6	0	3.2e-03	0.41

We separately crossed heterozygous *Celf1* ^+/−^ mice from two different inbred strains (129SvPas and C57BL/6N) to obtain F1 litters. We genotyped pups found dead after birth, and pups alive at 8–10 dpp. We show here the number of −/− and other (+/+ and +/−) pups. P-values for agreement with the Mendelian ratio (1/4 of −/− pups) were calculated by chi^2^ test for goodness of fit taking into account either only the live pups or all the pups.

### Spermiogenesis Blockage in *Celf1* ^−/−^ Mice During the First Wave of Spermatogenesis

**Figure 1:**
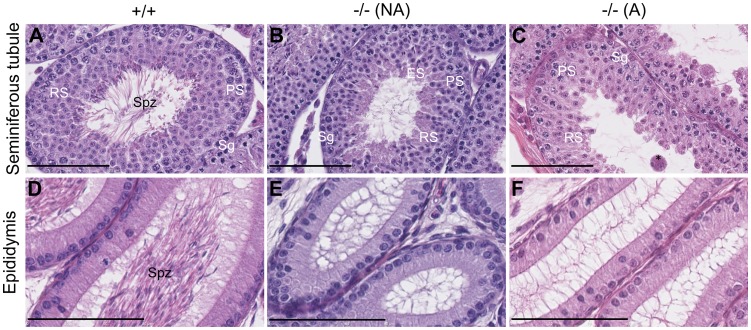
Incompletely penetrant blockage of spermiogenesis in Celf1^−/−^ mice during the first wave of spermatogenesis. Representative histological sections of testes of +/+ (left panels) and −/− (two right panels) mice at 42 days post-partum (dpp). **A–C**, seminiferous tubules. **D–F**, epididimes of the same respective mice. Depending on the presence of elongated spermatids in seminiferous tubules, KO mice were classified as “non affected” (**B**, **E**) or “affected” (**C**, **F**). Sg, Spermatogonia; PS, Pachytene Spermatocyte; RS, Round Spermatid; ES, Elongated Spermatid; Spz, Spermatozoa; *, multinuclear giant cell. Scale bars, 100 µm (**A–C**), 80 µm (**D–F**).

To investigate if the spermiogenesis blockage found in adult mice [18] already occurs during the first wave of spermatogenesis, we compared sections of seminiferous tubules from prepubertal +/+ and −/− mice. As expected for 42 dpp males [19], tubules and epididimes from +/+ mice contained spermatozoa (Figures 1A, 1D). The tubules from −/− animals either contained elongated spermatids (Figure 1B), revealing spermiogenesis set-up, or only contained round spermatids (Figure 1C), suggesting a spermiogenesis blockage. The tubules devoid of elongated spermatids contained multinuclear giant cells (Figure 1C), possibly as a consequence of the spermiogenesis blockage as observed in adults [18]. The ratio between mice with tubules containing or not elongated spermatids was roughly 1∶1. Hence, the first wave of spermatogenesis is blocked at the round spermatid stage in about half of the −/− mice.

### The First Wave of Spermatogenesis is Delayed in *Celf1* ^−/−^ Mice

**Figure 2:**
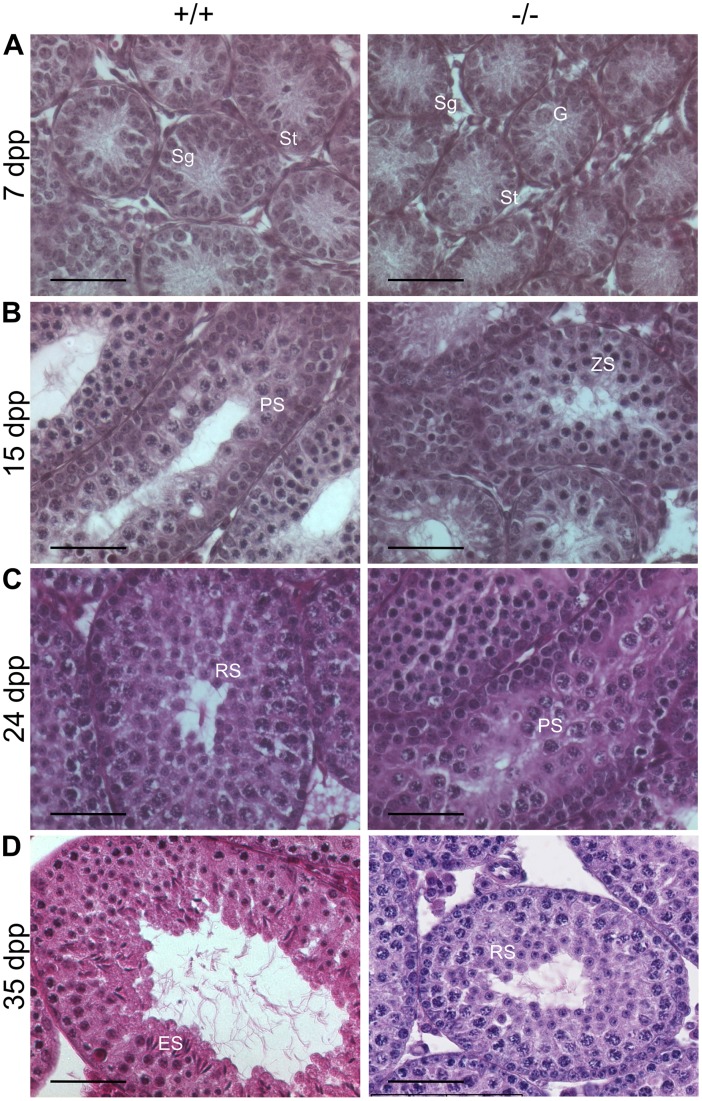
The first wave of spermatogenesis is delayed in Celf1^−/−^ mice. Representative histological sections of testes of homozygous *Celf1* ^+/+^ mice (left panels) and *Celf1* ^−/−^ mice (right panels) at different prepubertal ages. **A**, 7 dpp; **B**, 15 dpp; **C**, 24 dpp; **D**, 35 dpp. G, Gonocyte; Sg, Spermatogonia; St, Sertoli cell; ZS, Zygotene Spermatocyte; PS, Pachytene Spermatocyte; RS, Round Spermatid; ES, Elongated Spermatid. Scale bars, 50 µm.

**Figure 3:**
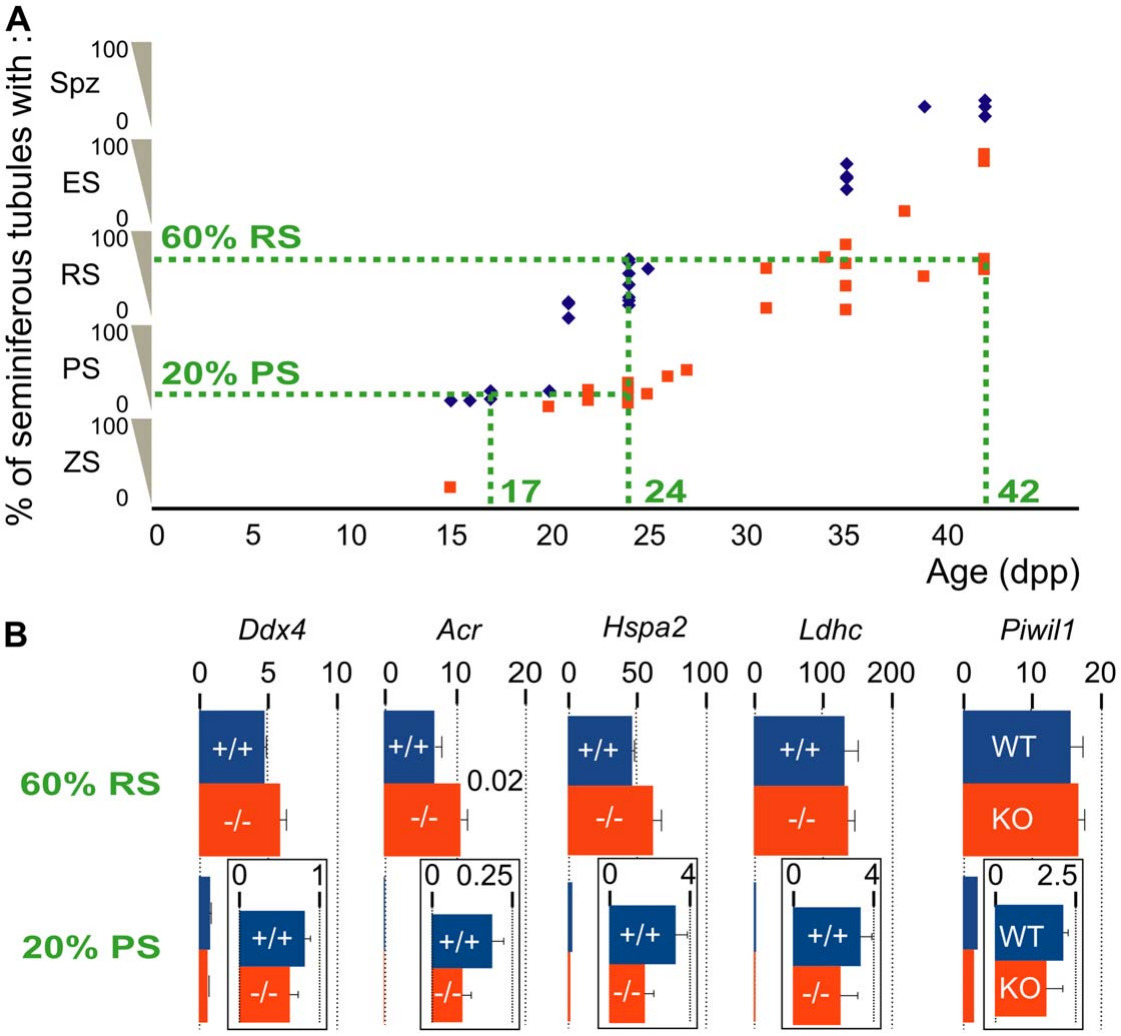
Quantification of the delay of the first wave of spermatogenesis. A , For each mouse, we analysed 300 seminiferous tubules in 15 testis sections. We classified each tubule according to the most differentiated germ cells that it contained (ZS, Zygotene Spermatocyte; PS, Pachytene Spermatocyte; RS, Round Spermatid; ES, Elongated Spermatid; Spz, Spermatozoa). Next, we calculated for each mouse the percentage of seminiferous tubules of each class, and we plotted the percentage of seminiferous tubules of the most advanced class against the age. Blue diamonds and orange squares correspond to individual *Celf1* ^+/+^ and *Celf1* ^−/−^ mice respectively. **B**, We quantified by real-time RT-PCR the relative amounts of the indicated mRNAs (Mouse Vasa Homolog, *Ddx4*; Proacrosine, *Acr*; Heat Shock Protein 70.2, *Hspa2*; Lactate Dehydrogenase 3, *Ldhc*; Miwi, *Piwil1*) for +/+ and −/− testes of similar stages of spermatogenesis but different ages: lower panels and inserts, 17 dpp +/+ (blue bars) and 24 dpp −/− (orange bars) testes with 20% of seminiferous tubules of the PS class; upper panels, 24 dpp +/+ (blue bars) and 42 dpp −/− (orange bars) testes with 60% of seminiferous tubules of the RS class. Results are expressed as the means of 3–5 animals for each genotype. Error bars are standard deviations. We used a Student’s t-test to statistically compare the +/+ and the −/− genotypes, and the p-values below 0.1 are given on the right of the corresponding bars.

Although the 42 dpp −/− mice were splited between those that encountered spermiogenesis during the first wave of spermatogenesis and those that did not, we observed that none of the 42 dpp −/− seminiferous tubules contained spermatozoa, the last stage of spermiogenesis (Figure 1B–C). Consistently, −/− epididimes were empty, contrasting with +/+ epididimes that started accumulating spermatozoa at this age (Figures 1 D–F). This suggests that the first wave of spermatogenesis is delayed in −/− animals. To characterize that delay, we compared histological sections of testes from younger mice. Representative photographs are shown in Figure 2. At 7 dpp, +/+ and −/− seminiferous tubules were mainly composed of Sertoli cells (St) and spermatogonia (Sg) at the periphery (Figure 2A). However, gonocytes (G, foetal germ cells) were found in 7 dpp −/− testes (right panel), but not in 7 dpp +/+ testes (left panel). At 15 dpp, meiosis reached the pachytene spermatocyte (PS) stage in +/+ animals whereas the most differentiated cells in −/− mice were zygotene spermatocytes (ZS, Figure 2B). At 24 dpp, round spermatids were present in the seminiferous tubules of +/+ mice (Figure 2C, left panel), demonstrating the completion of meiosis. We only observed pachytene spermatocytes in −/− mice at the same age (Figure 2C right panel). The ages at which the first round spermatids were observed were 21 dpp in +/+ animals and 31 dpp in −/− mice (see Figure 3A). In 35 dpp +/+ mice, we observed elongated spermatids in all the seminiferous tubules (Figure 2D left panel). By contrast, the −/− tubules at the same age only harboured round spermatids as the most advanced stages (Figure 2D right panel).

The above data suggest that, compared with *Celf1* ^+/+^ mice, the onset of the first wave of spermatogenesis is markedly delayed in *Celf1* ^−/−^ mice. To obtain a quantitative view of this delay, we analysed histological sections of testes of several +/+ and −/− mice and, for each testis section, we determined the most advanced stage of spermatogenesis. Next, we measured within each testis the percentage of seminiferous tubules that contained germ cells at that most advanced stage, and we plotted it against the age (Figure 3A). The first wave of spermatogenesis was as previously described in +/+ animals [19], and was delayed in all the −/− mice. This delay was on average 7 days in young mice. For example, 20% of the seminiferous tubules of +/+ mice taken around 17 dpp contained pachytene spermatocytes, whereas this value was only observed in −/− mice around 24 dpp. The delay increased with the age of the mice, as 60% of seminiferous tubules contained round spermatids around 24 dpp in +/+ animals but only at 31 to 42 dpp in −/− animals. As previously noticed (Figures 1B–C), the most advanced stage of germ cell differentiation in 42 dpp −/− testes was either round or elongated spermatids. Noteworthingly, all the −/− testes older than 31 dpp contained round spermatids. This confirms that the 42 dpp −/− testes devoid of elongated spermatids suffer from a blocked spermiogenesis, and not from a longer delay of germ cell differentiation. In contrast to 42 dpp −/− testes, the testicular contents of different *Celf1* ^−/−^ mice up to 35 dpp were homogeneous, and it was not possible to predict, among the 35 dpp mice, which ones would have supported spermiogenesis if they had been allowed to grow older. Together, these data show that the first wave of spermatogenesis is delayed by several days in −/− mice in a fully penetrant manner, while spermiogenesis is blocked during the first wave in −/− mice in an incompletely penetrant manner.

We next asked if the first wave of spermatogenesis differed in −/− and +/+ mice by additional criteria. To do this, we compared the expression of several germ cell markers previously shown to be down-regulated in adult −/− testes [18]. Preliminary experiments revealed significantly reduced expression levels of these markers in prepubertal −/− testes as compared with +/+ testes taken at the same age (data not shown), but these results might simply be due to the delayed testicular development. Hence, we measured the expression of these markers in +/+ and −/− testes that had reached similar stages of spermatogenesis. On the one hand we compared 17 dpp *Celf1* ^+/+^ with 24 dpp *Celf1* ^−/−^ animals, where 20% of seminiferous tubules contained pachytene spermatocytes as the most advanced stage (see Figure 3A). On the other hand, we compared 24 dpp *Celf1* ^+/+^ males with 42 dpp *Celf1* ^−/−^ males (devoid of elongated spermatids) where 60% of seminiferous tubules contained round spermatids (see Figure 3A). Except for *Acr* in testes where 60% of seminiferous tubules contained round spermatids, none of the tested germ cell markers was expressed at different levels in −/− and +/+ males (Figure 3B). This contrasts with the situation in adults where *Hspa2*, *Ldhc* and *Piwil1* are down-regulated in −/− testes [18]. This suggests that, except for the delay and the spermatogenesis blockage in part of the animals, *Celf1* inactivation does not dramatically affect gametogenesis during the first wave of spermatogenesis.

### The Setting-up of Steroidogenesis is Delayed in *Celf1* ^−/−^ Mice

**Figure 4:**
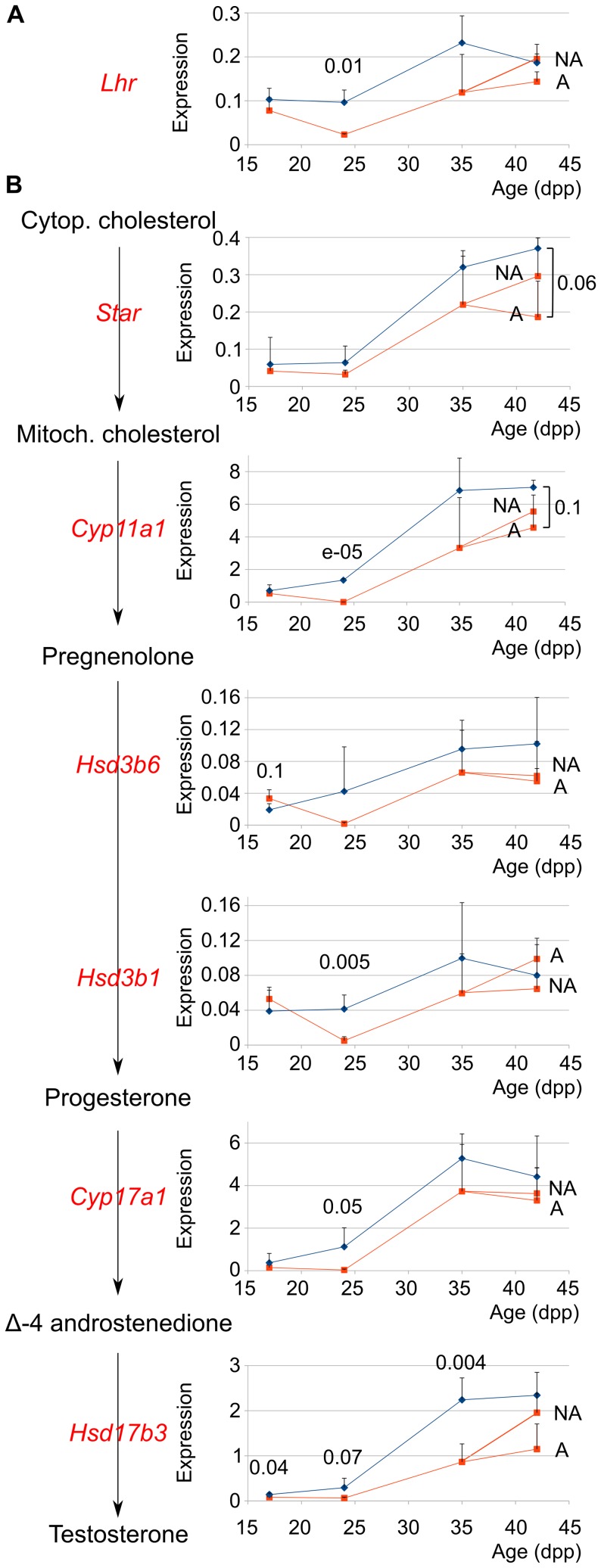
The expression of Leydig cells markers is delayed in prepubertal *Celf1* ^−/−^ mice. We quantified by real-time RT-PCR the relative amounts of the indicated mRNAs in +/+ (blue diamonds) and −/− (orange squares) testes at the indicated prepubertal ages. NA (not affected) and A (affected) refer to 42 dpp −/− mice with and without elongated spermatids respectively based on histological analyses. **A**, *Lhr.* **B**, The main pathway of steroidogenesis in rodents and the corresponding enzymes [37]. *Hsd3b1* and *Hsd3b6* have different expression patterns but encode enzymes with similar activities [22]. Results are expressed as the means of 3–5 animals for each age and genotype. Error bars are standard deviations. We used a Student’s t-test to statistically compare the different genotypes of identical ages, and we show the p-values below 0.1 above the corresponding symbols.

The delay of gametogenesis in −/− mice may be specific for germ-cells differentiation, but it may also reflect a more general delay of testicular development. To discriminate between these possibilities, we compared in +/+ and −/− prepubertal mice the expression of the *Lhr* gene that encodes the luteinizing hormone receptor (Figure 4A), and of steroidogenic enzymes that metabolize cholesterol into testosterone in several steps (Figure 4B). The expressions of these genes are markers of Leydig cells maturation and functions. They increased with the age of the animals both in +/+ and −/− males but with different kinetics. Between 17 and 24 dpp, the *Cyp11a1*, *Hsd3b6* and *Cyp17a1* mRNAs began accumulating in +/+ animals, whereas they remained at low levels in −/− animals. All the tested genes were stimulated between 24 and 35 dpp irrespective of the genotype. We classified 42 dpp −/− mice as supporting or not spermiogenesis (see above), and we analysed separately these two classes. In −/− animals, the expression of 5 genes about 7 (*Lhr*, *Star*, *Cyp11a1*, *Hsd3b1* and *Hsd17b3)* was stimulated between 35 dpp and at least one class of 42 dpp mice, whereas it was the case for only 1 gene (*Star*) in +/+ animals. Together, these data show that genes expressing steroidogenic enzymes start being expressed and reach a plateau earlier in +/+ than in −/− mice, demonstrating that the setting-up of steroidogenesis is delayed in −/− males. However, at 42 dpp, the difference of expression between +/+ and affected −/− mice is weakly significant for only two genes, *Star* and *Cyp11a1*, showing that the tested genes are less differentially expressed at 42 dpp than at earlier ages. This suggests that −/− mice catch up their delay by the end of prepubertal life.

We compared more thoroughly the two classes of 42 dpp −/− males (Figure 4). Four genes (*Lhr*, *Cyp11a1*, *Hsd3b6* and *Cyp17a1*) were expressed at very similar levels. Among the three other genes, two (*Star* and *Hsd17b3*) were possibly more strongly expressed in testes with elongated spermatids than in testes without elongated spermatids while the third one (*Hsd3b1*) was potentially more strongly expressed in testes without elongated spermatids. There is therefore no correlation between the capacity of −/− testes to fulfil spermiogenesis and the expression of Leydig cells markers taken as a whole.

## Discussion

Previously we have shown that *Celf1* ^−/−^ mice are barely viable, with about one-third of mice on a mixed 129SvPas*C57BL/6N background being alive 8–10 days after birth [18]. Here, we have been unable to identify any live *Celf1* ^−/−^ mice on 129SvPas or C57BL/6N backgrounds. A similar genetic background-dependent viability has been described for other gene disruptions in mice. For example, homozygous inactivation of *Hsf1* is virtually lethal in a 129Sv background, but not in other, mixed backgrounds [20]. Consequently, *Celf1* ^−/−^ mice can be analysed only on a mixed background, and this is probably at the origin of a high phenotypic variability. Indeed, the different traits of −/− mice have different penetrances. During the first wave of spermatogenesis, all the −/− mice show a delayed gametogenesis and a delayed expression of Leydig cells markers and steroidogenic enzymes, but spermiogenesis is only blocked in a fraction of them. We have previously shown that spermiogenesis is also blocked in a fraction of adult males [18]. Hence, fully penetrant traits are the delayed spermatogenesis and steroidogenesis in prepubertal animals, while an incompletely penetrant trait is the spermiogenesis arrest during the first wave of spermatogenesis and in adults. Spermatogenesis is highly dependant on testosterone [21]. Hence, delayed steroidogenesis might be a major cause for delayed gametogenesis, and the observation that these traits both are completely penetrant is consistent with this hypothesis. However, there are probably additional causes for the delay of germ cell differentiation as it is already observed at 7 dpp, before the onset of steroidogenesis [22].

Another fully penetrant trait of −/− mice is their reduced size [18], and this raises the question of a potential link between CELF1, delayed steroidogenesis and spermatogenesis, and reduced size. In humans, there is apparently no large-scale correlation between the size at birth, the prepubertal and postpubertal growth and the age of puberty in boys [23]. However, in particular genetic backgrounds, relationships exist between growth and onset of puberty. The Laron syndrome is a dwarfism due to a mutation is the Growth Hormone (GH) receptor. Sexual maturation is delayed in men with Laron syndrome [24], whereas for boys with a homozygous deletion of exon 3 (a gain-of-function mutation) pubertal onset is at a younger age [25]. In model animals, puberty is also delayed in male mice inactivated for GH receptor gene [26], and this may be due to a delayed maturation of Leydig cells, as GH stimulates the maturation of Leydig cells, both directly [27] and through IGF-1 [28]. Futhermore, according to a systematic analysis of gene expression in human [29], CELF1 is virtually ubiquitously expressed, including in the pituitary gland and the hypothalamus. Hence, it is tempting to speculate that the homozygous inactivation of *Celf1* results in fully penetrant defects in the GH/IGF-1 pathway that would reduce body size and delay puberty by delaying the maturation of Leydig cells. and testing this hypothesis will require additional experiments.

A spermiogenesis blockage has been reported in mice that suffer from impaired Leydig cells functions due to an inactivation of the LH receptor gene [30], [31] or androgen receptor gene in Leydig cells [32]. It is therefore tempting to hypothesize a supplemental link between Leydig cells dysfunctions and spermatogenesis defects in −/− males: not only delayed Leydig cells maturation would cause delayed gametogenesis during the first wave, but defective Leydig cells functions would block spermiogenesis in part of the −/− mice. We found no global correlation between the expression of steroidogenic enzymes and the capacity of −/− testes to fulfil spermiogenesis during the first wave, ruling out that a major dysfunction of Leydig cells would be the cause of the spermiogenesis blockage. However, *Star* and *Hsd17b3* tended to be more strongly expressed in 42 dpp −/− testes with elongated spermatids (Figure 4), and other genes involved in Leydig cells functions are also probably differentially expressed in −/− testes supporting or not spermiogenesis. The spermatogenesis defects in part of the *Celf1* ^−/−^ mice may therefore be due to subtle defects of Leydig cells that could be investigated by systematic approaches.

Germ cell markers are down-regulated in adults with a blocked spermiogenesis [18], but not in prepubertal −/− mice compared with wild-type mice of similar development (Figure 3B). In adults, intercellular cross-talks maintain a critical cell ratio between the different germ cell stages, but these cross-talks are not set up during the first wave of spermatogenesis [33]. We can therefore propose a hypothetical link between the occurence of cross-talks and the down-regulation of germ cell markers. Specifically, the down-regulation probably reflects a partial depletion rather than a dysfunction of germ cells, because these genes are differently regulated and the corresponding gene products are involved in different molecular pathways. Consequently, the fall in number of elongated spermatids due to spermiogenesis blockage in −/− mice may reduce the number of more immature germ cells owing to intercellular cross-talks in adults, but not during the first wave. Hence, the inactivation of *Celf1* would reduce the amount of germ cells in adults as a consequence of the spermiogenesis blockage rather than through a direct effect on cell proliferation.

## Materials and Methods

### Ethics Statements

Animals were bred in the Biosit animal facilities as approved by the French animal care agency (Direction des Services vétérinaires, approval number A3523840). Experiments were made according to standard procedures after acceptance by the local ethics commitee (Comité rennais d’éthique en matière d’expérimentation animale, approval number R-2011-CGC-01).

### Animals

The tm1Cba allele of the *Celf1* gene was shown to be null [18] and is noted - (minus) throughout this manuscript. This allele is present on three genetic backgrounds. The mixed background (C57BL/6N*129SvPas) has been described [18]. We obtained backgrounds congenic to 129SvPas and C57BL/6N strains by more than 10 backcrosses. We intercrossed heterozygous mice to obtain *Celf1* ^−/−^ animals and we genotyped them at 8–10 dpp by PCR on tail tips as described [18].

For each animal one testis associated with the epididymis was removed and fixed during four to six hours at RT in Bouin’s fluid, dehydrated and embedded in paraffin wax. Sections of 7 µm were made and stained with hematoxylin and eosine (Shandon). The other testis was snap frozen into liquid nitrogen and crushed in Trireagent (Euromedex. 1 ml Trireagent/testis). RNA was recovered according to the manufacturer instructions and quantified by spectrophometry (Nanodrop).

### Real-Time RT-PCR

Reverse transcription was made following standard procedures using random primers and Superscript II reverse transcriptase (Invitrogen), except for “RT-” controls where the enzyme was omitted. We made real-time PCR with an ABI Prism 7900 device (Applied) using SybrGreen mastermix and the primers given below. For each mRNA sample from individual testes, quantifications were made in triplicate. We checked that the RT- controls gave no amplification, or at a Ct far above that obtained with the corresponding RT+ conditions. Relative mRNA quantities were given by the difference of the Ct with the Ct of *Hprt* gene according to the formula [relative quantity = 2exp(CtHprt-Ctgene)], or by a double normalisation with *Hprt* and Beta-2 microglobulin (*B2m*) genes as described [34].

Primer sequences for *Acr* (D00754), *Ldhc* (X04752), *Hspa2* (BC052350), *Piwil1* (NM_021311), *Gapdh* (XM_354654), *Lhr* (M81310), *Cyp11a1* (AF195119) and *Hsd17b3* (U66827) have been published [18], [22], [35], [36]. The sequences of the other primers are:

*Ddx4* TACCTATGTGCCTCCCAGCTTC and TGTATTCAACGTGTGCTTTGCC;

*Hprt* CTGGTGAAAAGGACCTCTCG and TCAAGGGCATATCCAACAACAAAC;

*B2m* TGGTGCTTGTCTCACTGACC and CCGTTCTTCAGCATTTGGAT;

*Hsd3b6* TCCCCATTCAGAGCATGTATAGC and TTTTTTTGAGGTATTGACAAGTATTTATTG;

*Hsd3b1* CTCAGTTCTTAGGCTTCAGCAATTAC and CCAAAGGCAGGATATGATTTAGGA.
